# Testing the Activity of Complement Convertases in Serum/Plasma for Diagnosis of C4NeF-Mediated C3 Glomerulonephritis

**DOI:** 10.1007/s10875-016-0290-5

**Published:** 2016-05-05

**Authors:** Anna M. Blom, Fernando Corvillo, Michal Magda, Grzegorz Stasiłojć, Pilar Nozal, Miguel Ángel Pérez-Valdivia, Virginia Cabello-Chaves, Santiago Rodríguez de Córdoba, Margarita López-Trascasa, Marcin Okrój

**Affiliations:** Department of Translational Medicine, Lund University, 20502 Malmö, Sweden; Immunology Unit, University Hospital La Paz, IdiPAZ, Madrid, Spain; Servicio de Nefrología Hospital Universitario Virgen del Rocío, Sevilla, Spain; Centro de Investigaciones Biológicas, Consejo Superior de Investigaciones Científicas (CIB-CSIC), Centro de Investigación Médica en Red (CIBERER U738), Madrid, Spain; Unit 754, Centre for Biomedical Research on Rare Diseases (CIBERER), Madrid, Spain; Department of Medical Biotechnology, Intercollegiate Faculty of Biotechnology UG‑MUG, Medical University of Gdańsk, Dębinki 1 street, 80-210 Gdańsk, Poland

**Keywords:** Complement system, complement convertase, C4NeF, C3 glomerulonephritis

## Abstract

Autoantibodies termed C3-nephritic factor (C3NeF), which stabilize convertases of the alternative complement pathway, often stimulate autoinflammatory diseases. However, knowledge about analogous autoantibodies acting on the classical pathway (C4NeF) is limited to a few reports, which indicate association with kidney dysfunction, systemic lupus erythematous, and infections. C4NeF may appear independently from C3NeF, but the lack of a routine diagnostic method predisposes C4NeF for being an underestimated player in autoinflammatory episodes. We tested the activity of classical convertases directly in serum/plasma to screen samples from 13 patients with C3 glomerulopathies and identified one patient showing significantly prolonged half-life of these enzymes. Observed effect was reproduced by immunoglobulins purified from patient’s plasma and additionally confirmed on classical convertase built from purified components. Isolated immunoglobulins protected classical convertases from both spontaneous and inhibitor-driven decay but not from C4b proteolysis. The patient had a decreased serum level of C3, elevated sC5b-9, and normal concentrations of factor B and C4. Neither C3NeF nor other autoantibodies directed against alternative pathway proteins (factor H, factor B, factor I, C3, and properdin) were found. Genetic analysis showed no mutations in *C3*, *CFB*, *CFH*, *CFI*, *MCP*, *THBD*, and *DGKE* genes. Renal biopsy revealed a membranoproliferative pattern with intense C3 deposits. Our results underline the importance of C4NeF as an independent pathogenic factor and a need for the implementation of routine examination of classical convertase activity. Proposed method may enable robust inspection of such atypical cases.

**Electronic supplementary material:**

The online version of this article (doi:10.1007/s10875-016-0290-5) contains supplementary material, which is available to authorized users.

## Introduction

Three different routes may drive the complement system, an important constituent of innate immunity: the classical, lectin, and alternative pathways. Whereas the first two pathways are initiated by certain stimuli such as antibodies bound to the cell surface or the presence of pathogen-specific sugar moieties, the alternative pathway is constantly active at a low level and its further propagation depends on the lack of restriction by endogenous complement inhibitors [1]. The alternative or classical/lectin pathway complement convertases are the elements of the cascade, which are particularly controlled by complement inhibitors. Otherwise, convertases produce opsonins, anaphylatoxins, and initiate the formation of the membrane attack complex aimed to lyse target cells [2]. Under physiological conditions, the maximal activity of convertases is reached relatively quickly after pathway initiation (seconds or minutes, depending on complement availability, as shown in [3]) and after this time their activity decreases due to spontaneous and inhibitor-driven decay as well as proteolysis of C3b and C4b components by factor I [4]. Diverse modes of complement activation determine the risk of autoinflammatory diseases resulting from misguided or uncontrolled complement attack. A loss of regulation of the alternative pathway is sufficient for pathologic events whereas in the case of the classical and lectin pathways, an analogous condition must be accompanied with a pathway-specific stimulus present on self cells. Indeed, mutations in alternative pathway components are well-known etiologic/risk factors for diseases such as C3 glomerulopathies (C3G) [5] or atypical hemolytic uremic syndrome (aHUS), which manifest already at a very young age [6]. Another condition, which leads to the unrestricted activity of alternative convertases, is the presence of autoantibodies termed C3NeF. C3NeF binds neoepitopes formed upon the assembly of the convertase and stabilizes it, resulting in consumption of C3 (hypocomplementemia) and amplification of downstream events in the complement cascade. Occurrence of C3NeF is reported in almost all patients with dense deposit disease and in half of the patients with C3GN [7]. Interestingly, this autoantibody was also found in an individual showing no disease symptoms [8] and its presence in some C3GN patients did not cause hypocomplementemia [9], but, on the other hand, the same study reported C3NeF-positive C3GN in the absence of other known disease-causing agents [9]. In contrast to the deregulation of alternative pathway convertases by either mutations in pivotal pathway components or autoantibodies, similar phenomena for the classical pathway convertases are poorly described, with not a single report about a gain-of-function mutation and only a few reports about C4NeF (analogous to C3NeF). Incidence of C4NeF was briefly reviewed in [10] and reported in postinfectious glomerulonephritis [11, 12], membranoproliferative glomerulonephritis [13], systemic lupus erythematous [14], and recently in a patient with sepsis caused by *Neisseria meningitidis* [15]. There are two reports on C4NeF occurrence in large cohorts with renal diseases, which show C4NeF in 19 out of 100 patients [13] and 19 out of 197 patients [16], respectively. Interestingly, the percentage of patients double-positive for C3NeF and C4NeF was 52 [13] and 10 % [16] depending on cohort, showing that these two kinds of activities may appear independently of each other. Also, there are reports showing that C4NeF may stabilize not only C3 classical convertase but also C5 classical convertase [15, 17]. There is no routine diagnostic procedure for C4NeF determination. Available experimental methods are based on multistep hemolytic assays performed on sheep erythrocytes coated with purified components of classical convertases (EAC142 or EAC1423) [13, 15] or precipitation of stabilized fluid-phase C4b2a complexes followed by detection by sandwich ELISA [18]. An obvious limitation of detection systems based on purified complement components is elimination of interactions with other components from autologous serum, which are normally present under physiological conditions and may influence convertase formation and stability. On the other hand, detection of stabilized, fluid-phase classical convertase precipitated from patient serum does not give any information about enzymatic activity. We have designed a new method for assessment of convertase activity directly in patient’s serum or plasma, which makes use of C5 blockers: OmCI or eculizumab [19]. Thereafter, we showed that our approach enables proper detection of clinical samples with altered function of alternative convertases caused by either autoantibodies (C3NeF, anti-factor H) or mutations in complement proteins (C3, factor B) [19]. Herein, we report the usage of this method for screening for abnormally prolonged activity of classical convertases and, by doing so, identification of C4NeF activity in a patient with C3 glomerulonephritis of previously unknown etiology. Further analysis revealed that the specific activity responsible for the phenotype was conferred in the Ig fraction isolated from plasma.

## Materials and Methods

### Reagents, Sera and Patient Material

Normal human serum (NHS) was prepared from blood of healthy volunteers after written informed consent had been obtained and according to the permit by the ethics committee in Lund (permit number 2013/846). Blood was collected and kept at room temperature for 30 min to coagulate, then on ice for another 60 min followed by centrifugation for 7 min at 700×*g*. The serum fraction was collected, centrifuged again to remove residual erythrocytes, aliquoted, and stored at −80 °C until use. Thirteen patients with biopsy-proven C3 glomerulopathy (3 with dense deposit disease and 10 with C3 glomerulonephritis) diagnosed between 1970 and 2015 were selected based on the availability of sera and/or plasma samples to complete all assays, as well as histopathologic data (light microscopy, immunofluorescence, and electron microscopy) to review the pathologic diagnosis. Serum and EDTA-plasma samples were obtained under standard conditions with informed consent and with the approval of Hospital Universitario La Paz; blood was collected into plain tubes, allowed to clot at room temperature, and centrifuged for 10 min at 4 °C. After that, serum and plasma were collected, aliquoted, and kept frozen at −80 °C until use. List of the patients with indicated diagnoses and complement-related parameters is given in Table 1. Purified human C2, C3, C4, C4b, FB, FD, properdin, FI, and FH were purchased from Comp Tech. OmCI, a recombinant inhibitor of C5 cleavage, was expressed and purified as described in [19]. Guinea pig serum was purchased from Harlan Laboratories. Human sera depleted from C3 and/or C5 as well as mouse anti- C4d antibody (A253) were purchased from Quidel. Soluble CD55 (DAF) was produced recombinantly in eukaryotic system as described [20]. Factor I (FI) [21] and C4b-binding protein (C4BP) [22] used in C4b degradation assay were purified from human plasma. Monoclonal antibody MK104 against C4BP was homemade and purified using protein A affinity chromatography as described [23].

**Table 1:** Complement-related parameters of patient sera

Patient number	Diagnosis	C3 (mg/mL)	C4 (mg/mL)	FB (mg/mL)	Properdin (μg/mL)	C5 (mg/mL)	sC5b-9 (mg/L)	C3NeF	Alternative pathway autoantibodies	Genetic variations
1	DDD	0.09	0.24	N/A	29.3	0.084	N/A	+	–	
2	C3GN	0.06	0.20	0.15	21.3	0.013	0.9	–	–	C3 (c.1656G > C(HET), p.Trp552Cys)
3	DDD	0.20	0.17	0.27	24.6	0.016	N/A	+	N/A	
4	C3GN	0.04	0.34	0.39	17.5	0.045	3.0	–	Anti-FB	
5	DDD	0.56	0.26	0.13	15.1	0.107	N/A	–	Anti-FI	
6	C3GN	0.53	0.27	0.20	13.6	0.099	0.8	–	Anti-C3, FB, properdin and FI	CFH (c.76 T > C(HET), ENST00000367429, 5′UTR)
7	C3GN	0.13	0.29	0.36	14.1	0.079	2.3	–	–	
8	C3GN	0.63	0.26	0.23	30.4	0.086	0.3	–	Anti-C3, FB and properdin	
9	C3GN	0.59	0.17	0.17	22.8	0.118	N/A	–	–	
10	C3GN	0.50	0.20	0.21	15.5	0.111	1.1	–	–	
11	C3GN	0.41	0.23	0.14	19.8	0.087	N/A	–	Anti-properdin	
12	C3GN	0.05	0.36	0.24	28.0	0.029	3.3	–	–	C3 (c.1269 + 1G > A(HET), intron 11)
13	C3GN	0.18	0.24	0.24	16.0	0.026	1.6	–	–	
Normal range		(0.75–1.35)	(0.14–0.60)	(0.06–0.33)	(17.5–45.5)	(0.046–0.226)	(<0.87)			

*N/A* not assessed

### Genotyping and Mutation Screening

Genomic DNA was extracted from peripheral blood using standard procedures. Exons of the *CFH*, *MCP*, *CFI*, *C3*, *CFB*, *THBD*, and *DGKE* genes were amplified from genomic DNA using primers derived from the intronic sequences as described [24–26]. Automatic sequencing was performed in an ABI3730 sequencer using a dye terminator cycle sequencing kit (Applied Biosystems). The analysis of the *D*_*CFHR3-CFHR1*_ polymorphism and genomic rearrangements in the *CFH*-*CFHRs* region were assessed by multiplex ligation-dependent probe amplification (MLPA) with the P236 A1 ARMD mix 1 (MRC-Holland, Amsterdam, Netherlands).

### Properdin, C3, C4, FB, C5, and sC5b-9 Quantification

Properdin levels were measured by ELISA as described in [27]. Serum levels of C3 and C4 were measured by nephelometry (Siemens Healthcare, Marburg, Germany). Serum FB levels were determined by ELISA using 100 ng/well of protein G (GE Healthcare) purified polyclonal goat IgG anti-human FB antibody (Calbiochem, #341272) as capture antibody and a monoclonal mouse anti-human Bb (A227, Quidel, San Diego, CA) as the detection antibody. C5 serum levels were determined following an ELISA described in [28]. Circulating sC5b-9 was analyzed using a commercially available assay (A029, Quidel).

### C3NeF Detection by ELISA

C3NeF detection in serum samples was performed as previously described [29] with several modifications. Briefly, two ELISA assays were performed: COSP (to measure the stabilizing-capacity of serum C3NeF) and COIgG (to detect the binding of C3NeF to the AP C3 convertase). In both assays, ELISA plates (Medisorp, Nunc) were coated with C3b (1 μg/ml in PBS, overnight, 4 °C). Convertase was formed by adding 50 μl test serum (1/50 in assay buffer), immediately followed by 50 μl of a mixture comprising FB (1 μg/ml) and FD (0.2 μg/ml) with properdin (0.5 μg/ml) in assay buffer. For COSP, a 1/500 dilution of a monoclonal anti-human Bb fragment (Quidel, #A227) was used to detect the presence of residual Bb fragments on the stabilized C3bBbP complex. A peroxidase-conjugated goat anti-mouse IgG (H + L) (Invitrogen) diluted 1:1000 was used as secondary antibody, and the reaction was developed with OPD. The COIgG assay was performed as described in [29] except that the assay buffer contained 1 mM of MgCl_2_ instead of NiSO_4_ in order to reduce background.

### Antibody Binding to Immobilized C3, FB, FI, FH, and Properdin

ELISA plates were coated with 100 ng/well of purified C3, FB, FI, FH, or properdin. Plates were blocked with PBS-BSA 3 % in case of FB and properdin, and with PBS-BSA 0.1 % in FI and C3. Serum samples were diluted in PBS-BSA 0.1 %, and binding of autoantibodies was detected with polyclonal anti-human IgG-HRP conjugated antibody (Jackson Immunoresearch) in ABTS substrate, as described in [30]. Factor H autoantibodies were analyzed as previously described in [31].

### Purification of Whole Ig Fraction

Whole Ig fraction was purified from 1 ml of EDTA-plasma of patient seven and 1 ml of normal human serum pooled from 13 healthy volunteers, by affinity chromatography on 5 ml protein A and protein G Sepharose Hi-Trap columns (GE Healthcare). Samples were loaded, the unbound fraction was washed with PBS buffer and then Ig eluted with 0.1 M glycine-HCl buffer pH 2.5. Fractions containing proteins were immediately neutralized with 1/10 volume of 1 M Tris-HCl pH 8.0, dialyzed against PBS, and concentrated using 10 kDa molecular weight cut-off value device (Vivaspin).

### Functional Assays for Activity of Classical Convertases

Activity of classical complement convertases was tested either in full serum/plasma or in a setting with whole Ig fraction purified from patients’ blood, as described in [19] and [3], respectively. Additional analysis testing the stability of convertases built up from purified components was performed on sheep erythrocytes (Hatunalab AB or BioMaxima) sensitized with anti-sheep erythrocyte antibody Amboceptor (Behring). Erythrocytes were prepared as described [3] and then incubated with 2.5 μg/ml of C1 and C4 for 10 min at 30 °C in DGVB veronal buffer. Afterwards, 2.5 μg/ml of C2 was added with various dilutions of whole Ig fraction isolated from patient seven or normal human serum. Incubation was performed for up to 90 min, and reaction was stopped at chosen time points. Erythrocytes were washed with ice-cold 40 mM EDTA-GVB buffer and centrifuged for 1 min at 1000×*g*. Pellets were resuspended in 50 μl of fresh 40 mM EDTA-GVB containing guinea pig serum diluted 1:40 and overlaid with additional 50 μl of DGVB. After 20 min of incubation at 37 °C, cells were centrifuged. Supernatant was collected and placed in a flat-bottom plate, and absorbance at 405 nm was measured with a microplate reader.

### Determination of C4d Content

C4d measurement aimed to determine the rate of C4b inactivation by FI-mediated cleavage was performed by sandwich ELISA assay using anti-neoepitope C4d antibodies raised in-house, as described in [32]. Samples containing 5 μg/ml C4b (Comp Tech) and 10 μg/ml of C4BP and FI and alternatively control or patient’s whole Ig fraction or 500 μg/ml of rabbit-anti C4c antibody (Dako, #Q0369) were incubated for 1 h and then loaded onto ELISA plate.

### Visualization of C4b Cleavage Pattern

Thirty microgram per milliliter of C4b (Comp Tech) and 100 μg/ml of C4BP were mixed and optionally supplied with 10 μg/ml of FI and 500 μg/ml of rabbit-anti C4c antibody. Mixture was incubated overnight at 37 °C, and then 3x Laemmli buffer was added and after boiling for 5 min samples were separated by 10 % SDS-PAGE. After electrophoresis gel was blotted onto PVDF membrane and signal from C4 fragments was developed with mixture of 1:1000 mouse anti-C4d (clone LP69, Abcam) and 1:1000 of goat anti-C4 (Comp Tech, #A205), followed by mixture of goat anti-rabbit and anti-mouse HRP-conjugated Abs (Dako, #P0448 and #P0447). Western blot was visualized with DAB, as described in [33].

## Results

### Screening of Serum/Plasma Samples for Prolonged Activity of Classical Complement Convertases

**Fig. 1:**
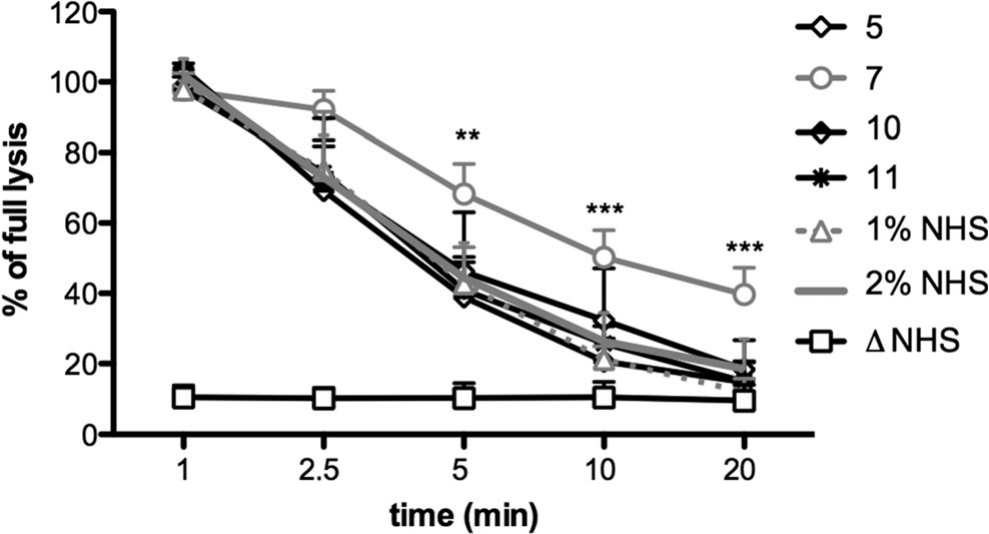
Screening of clinical serum and plasma samples for prolonged activity of classical convertases. Thirteen samples collected from the patients with C3 glomerulopathies were diluted to final concentration of 1 %, mixed with equal volume of NHS and inhibitor of C5 cleavage was added. Samples were incubated with sensitized sheep erythrocytes for indicated time period and then washed and incubated with guinea pig serum diluted in 40 mM EDTA-GVB buffer, which disabled de novo convertase formation but allowed development of lytic sites only from pre-existing convertases. Percentage of lysis measured at 405 nm was referred to the equal amount of sensitized erythrocytes lysed with equal volume of water. Data were collected from three independent experiments, statistical significance was assessed by two-way ANOVA at **p < 0.01 and ***p < 0.001, respectively. Only four clinical samples including #7 were shown for better clarity of the graph but none of the clinical samples but #7 showed statistically significant differences when compared to NHS controls. Graph showing all 13 patients versus healthy controls (normal human serum) is presented in Supplementary Fig. 1

The plasma/serum samples of 13 patients with confirmed C3 glomerulopathies (listed in Table 1) were included in a screening assay testing the activity of classical convertases over time (Fig. 1). Since the patients presented with different degrees of hypocomplementemia, all samples were diluted to 1 % final concentration and then mixed with an equal amount of normal human serum (NHS). This step enabled normalization of the readout but the addition of an extra 1 % of NHS did not affect convertase activity curves, as evidenced by the lack of differences between 1 and 2 % NHS samples (Fig. 1). However, it allowed the detection of factors present in individual serum/plasma samples which caused prolongation of classical convertase activity. In the case of sample 7, convertase activity was significantly elevated from the time point of 5 min (*p* < 0.01 according to two-way ANOVA, comparing to 1 % NHS) and continued to time points of 10 and 20 min (*p* < 0.001). The activity of other clinical samples did not differ significantly from NHS controls (Supplementary Fig. 1).

### Stabilization of the Classical C3/C5 Convertase Activity Is Supported by the Ig Fraction Isolated from the Patient’s Plasma

**Fig. 2:**
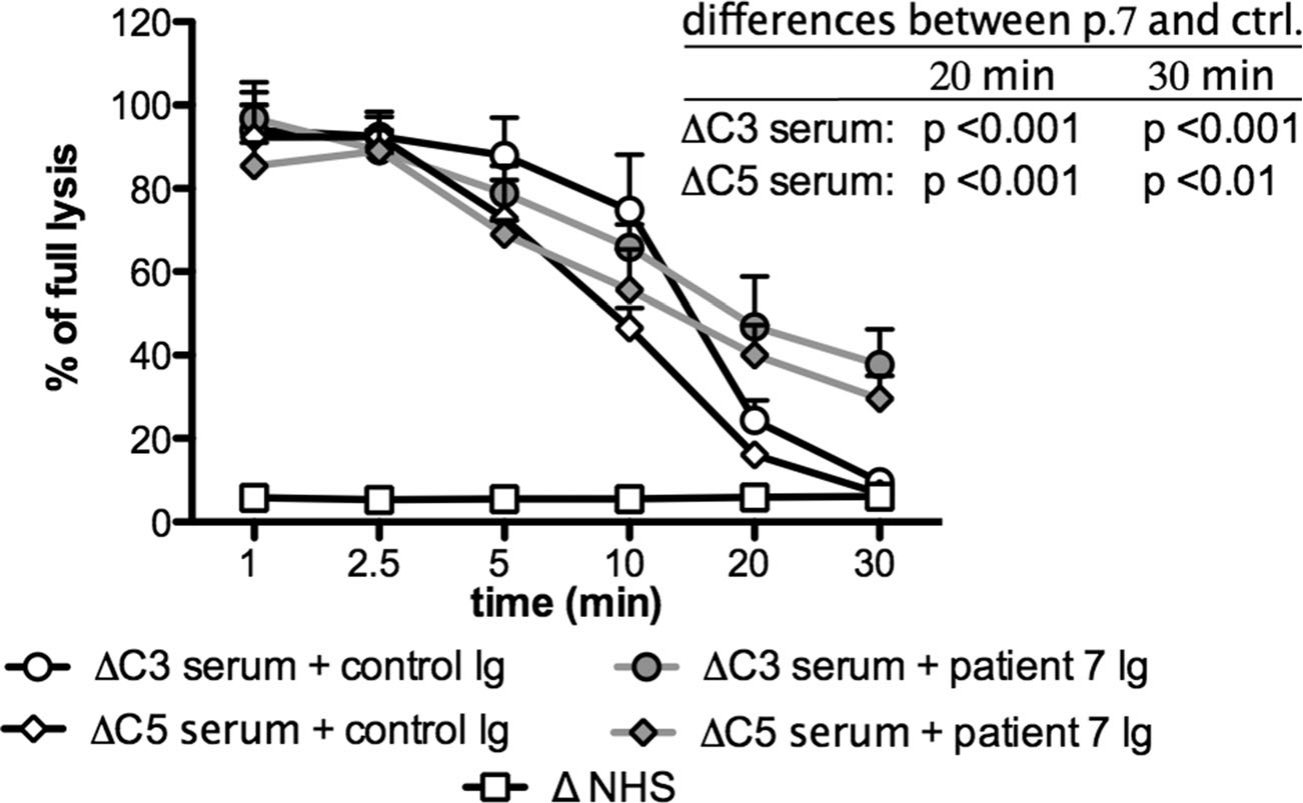
Influence of patient’s and control Ig on stability of C3 and C5 classical convertases. In order to assess the influence of total Ig fraction purified from patient seven plasma on classical C3 and C5 convertase, Ig preparations were mixed with 1 % C3-depleted or C5-depleted serum, respectively, and incubated with sensitized sheep erythrocytes for an indicated period of time. Samples were wzashed and incubated with guinea pig serum diluted in 40 mM EDTA-GVB buffer, which disabled de novo convertase formation but allowed development of lytic sites only from pre-existing convertases. Percentage of lysis measured at 405 nm was referred to the equal amount of sensitized erythrocytes lysed with equal volume of water. Ig preparation from NHS was used as a negative control. Data were collected from three independent experiments; statistical significance was assessed by two-way ANOVA

We investigated whether the effect on classical convertase exerted by plasma from patient seven could be reproduced by addition of the Ig fraction only to 1 % NHS. Total Ig was purified from 1 ml of the patient’s plasma by protein A/G affinity chromatography. The obtained preparation was concentrated to the initial plasma volume and used in an experiment similar to that presented in Fig. 1. Results showed no significant differences between NHS supplemented either with 1 % patient’s serum or an equal volume of purified Ig fraction (Supplementary Fig. 2). To confirm whether the prolonged stability of the classical complement convertase in the plasma of patient seven is attributed to C4NeF activity, we investigated whether purified antibodies from patient seven stabilize both C3 and C5 classical convertases. To answer this question, we applied another assay described in [3], which operates in C3- or C5-depleted serum. These sera support the formation of C3 and C5 classical convertases, respectively, on Ab-sensitized sheep erythrocytes. Addition of Ig purified from patient seven resulted in significantly prolonged activity of both classical convertases when compared to control Ig purified from NHS (Fig. 2).

### C4NeF from Patient Seven Prevents Both Spontaneous and Inhibitor-Driven Convertase Decay but Not Factor I-Mediated Proteolysis of C4b

**Fig. 3:**
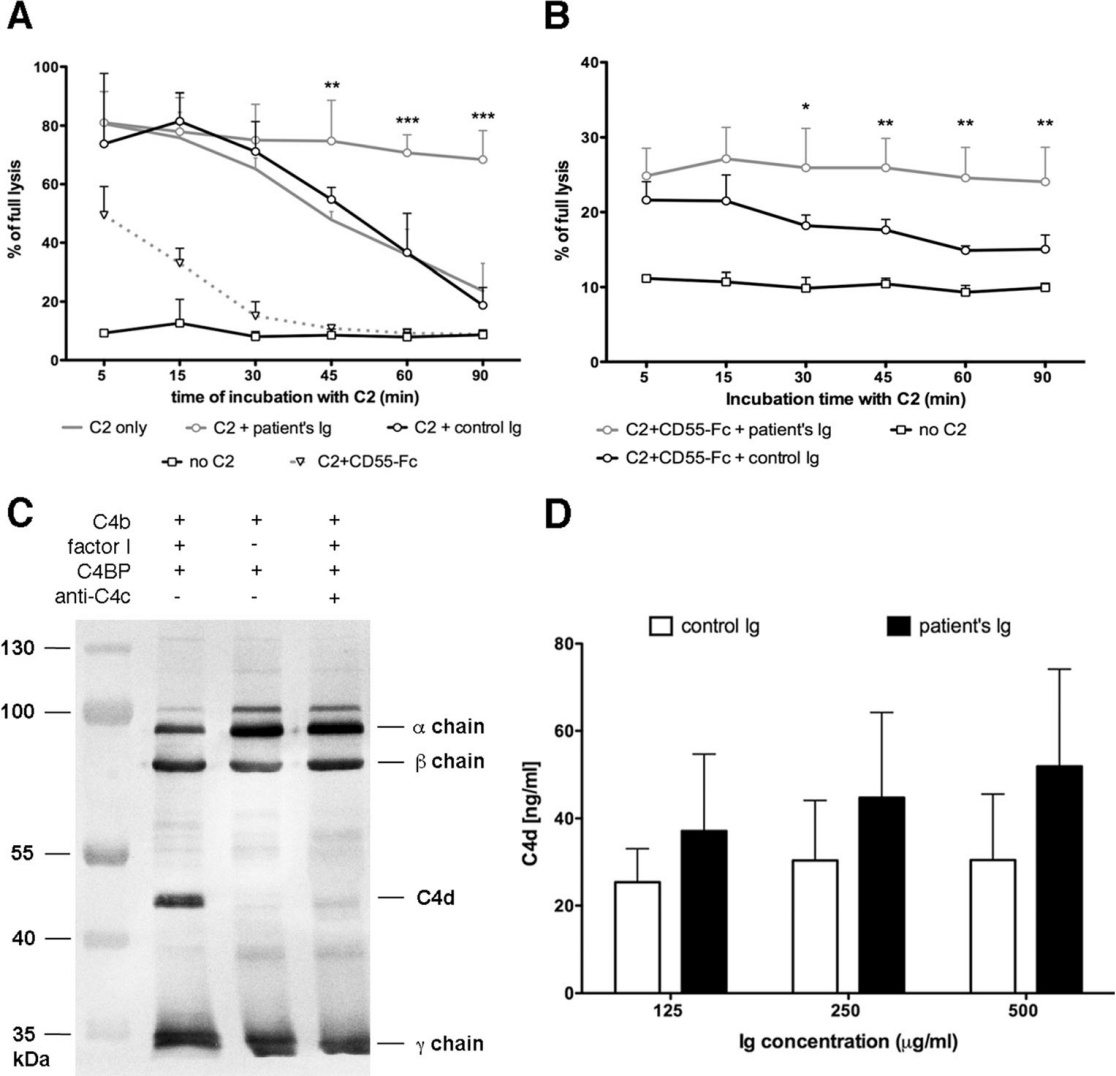
Mechanism of convertase stabilization by C4NeF isolated from patient seven. **a** Spontaneous decay of classical C3 convertase assembled from purified components. Sensitized erythrocytes were coated with C1 and C4. Addition of C2 initiated the process of convertase formation. Convertase activity was assessed at indicated time points in the same manner, as described in Figs. 1 and 2. **b** The same experiment as in **a** but with CD55-Fc added together with C2. **c** Representative blot of the C4b degradation pattern (C4b, factor I, and C4BP present) and comparison to negative control with no factor I added and sample, in which C4b cleavage was inhibited by excessive anti-C4c antibody. **d** Quantification of C4d produced upon C4b degradation in the presence of Igs isolated from patient seven or from NHS. When anti-C4c antibodies were tested in the same experiments, C4d readout was below the lower level of detection and therefore is not shown. All graphs show data collected from at least 3 independent experiments and statistical significance was assessed with two-way ANOVA assay at *p < 0.05, **p < 0.0,1 and ***p < 0.001

Further experiments aimed to identify the mechanism, by which C4NeF isolated from patient seven stabilizes classical convertases. First, we checked whether putative binding of autoantibodies to classical convertases protects them from spontaneous decay. Convertases were built up by deposition of purified C1 and C4 onto the surface of sensitized sheep erythrocytes followed by the addition of purified C2 together with Ig isolated from the patient or from NHS. Additional controls were aimed to test convertase decay, when either no Ig or a soluble version of complement inhibitor CD55 (CD55-Fc) were added. Erythrocytes incubated without C2 served as negative control. The addition of Ig isolated from NHS did not affect the convertase activity profile in contrast to the addition of Ig isolated from patient seven, which stabilized convertase activity (Fig. 3a). Addition of CD55-Fc resulted in diminished convertase activity at the first time point tested and a subsequent, rapid drop of activity down to the level of the negative control, as expected. Thereafter, we tested whether C4NeF from patient seven is capable of counteracting inhibitors which accelerate convertase decay, using the inhibitor CD55 as a model. Addition of C4NeF could not rescue the initial drop of convertase activity measured at the first time point, but in contrast to control Ig it efficiently prevented the accelerated drop in activity at further time points (Fig. 3b). Next, we checked whether C4NeF from patient seven also protected convertases from the FI-mediated cleavage of C4b. For example, antibodies targeting C4 may hinder binding sites for FI and/or its cofactors, and by doing so, disable C4b cleavage, as we showed in Fig. 3c for anti-C4c antibodies. Our preliminary experiment, in which we tested patient’s and control Igs in the same setting, did not show differences in C4b cleavage (not shown). However, reliable visualization of C4NeF influence on C4b cleavage with this method would demand substantial quantities of isolated C4NeF, which we could not supply. Therefore, we chose another technique based on the quantification of the C4d cleavage product by a highly sensitive sandwich ELISA [32], which allowed minimizing the amount of required C4b and so enabled the testing of a fair molar excess of antibodies over C4b. Surprisingly, there was a trend toward a more pronounced generation of C4d when the patient’s Ig were present but these differences did not reach statistical significance at any of the Ig concentrations tested (Fig. 3d). Nonetheless, based on these results, we conclude that the C4NeF activity of Ig isolated from patient seven could not be attributed to an interference with proteolytic inactivation of C4b. Using analogous method and function-blocking antibodies against FI and C4BP, we tried to assess the importance of convertase decay-acceleration versus FI-mediated proteolysis of C4b for overall convertase activity. Unfortunately, addition of purified IgG fraction isolated from the patients diminished trustful range of readout and prevented from reliable conclusions (not shown). Nonetheless, in previous publication, we showed that blocking of FI function does not affect stability of classical convertases as much as blocking of C4BP [3] and extrapolation of these findings seems to be applicable in the current case.

### Clinical Manifestation of Disease in Patient Seven

In order to analyze the case of patient seven, the only individual whose sample was yielding positive result in our C4NeF screening assay, we investigated related clinical data. He was a 55-year-old man referred to a hospital for a nephrotic syndrome study, on the basis of a casual finding of high proteinuria in a periodic analytical control. There was no familial history of renal disease, but he and his father and brothers are diagnosed with dyslipidemia and systemic arterial hypertension. At the time of the study, the patient presented normal blood cell counts, decreased total serum protein levels (48 mg/mL), as well as low serum albumin (2.8 mg/mL). IgG count was also low (4.23 mg/mL, normal range 7.25–19), but IgA and IgM were normal (1.53 mg/mL, normal range 0.5–3.4 and 0.83 mg/mL, normal range 0.45–2.8, respectively). Serum creatinine and urea were at the upper limit of normal range (12 and 400 mg/L). Hematuria and proteinuria (2 g/L) were found, reaching up to a protein loss of 16 g in 24-h urine analysis. Complement C3 serum levels were decreased, but C4 and C1q were within their normal ranges and factor B (FB) was even higher than normal range. A test for anti-nuclear autoantibodies was negative.

At this time, renal biopsy was performed. Of the 36 glomeruli obtained in the biopsy, three were totally sclerosed. Extracapillary proliferation was absent and sporadic leukocytic infiltrations were observed. Biopsy showed diffuse increased capillary walls, with split basal membrane and irregular subendothelial deposits. On direct immunofluorescence, intense clumpy C3 deposits on glomerular capillaries and mesangium were observed. IgG deposits showed a similar pattern but with lower intensity. Capillary and mesangial IgM, C1q, and C4d deposits were also evidenced, and kappa and lambda chains to a lesser extent. A complement related-glomerular disease was suspected, because of the membranoproliferative pattern with the intense C3 deposits observed in the biopsy and the persistently decreased C3 serum levels. Three months after diagnosis, renal function was progressively worsening, with serum creatinine of 13.7 mg/L and persistent nephritic syndrome; the patient was referred to perform additional, detailed complement studies. The decreased levels of circulating complement C3 and properdin suggested alternative pathway activation, but FB was not diminished. The patient had a high C5 convertase activity, which was reflected by a C5 concentration within lower levels of normal range and elevated sC5b-9. The patient was screened for the presence of C3NeF and other autoantibodies directed to alternative pathway proteins (FH, FB, FI, C3, and properdin), and all resulted negative. In the genetic analysis, no mutations were identified in *C3*, *CFB*, *CFH*, *CFI*, *MCP*, *THBD*, and *DGKE* genes.

### Technical Performance of the Assay

**Fig. 4:**
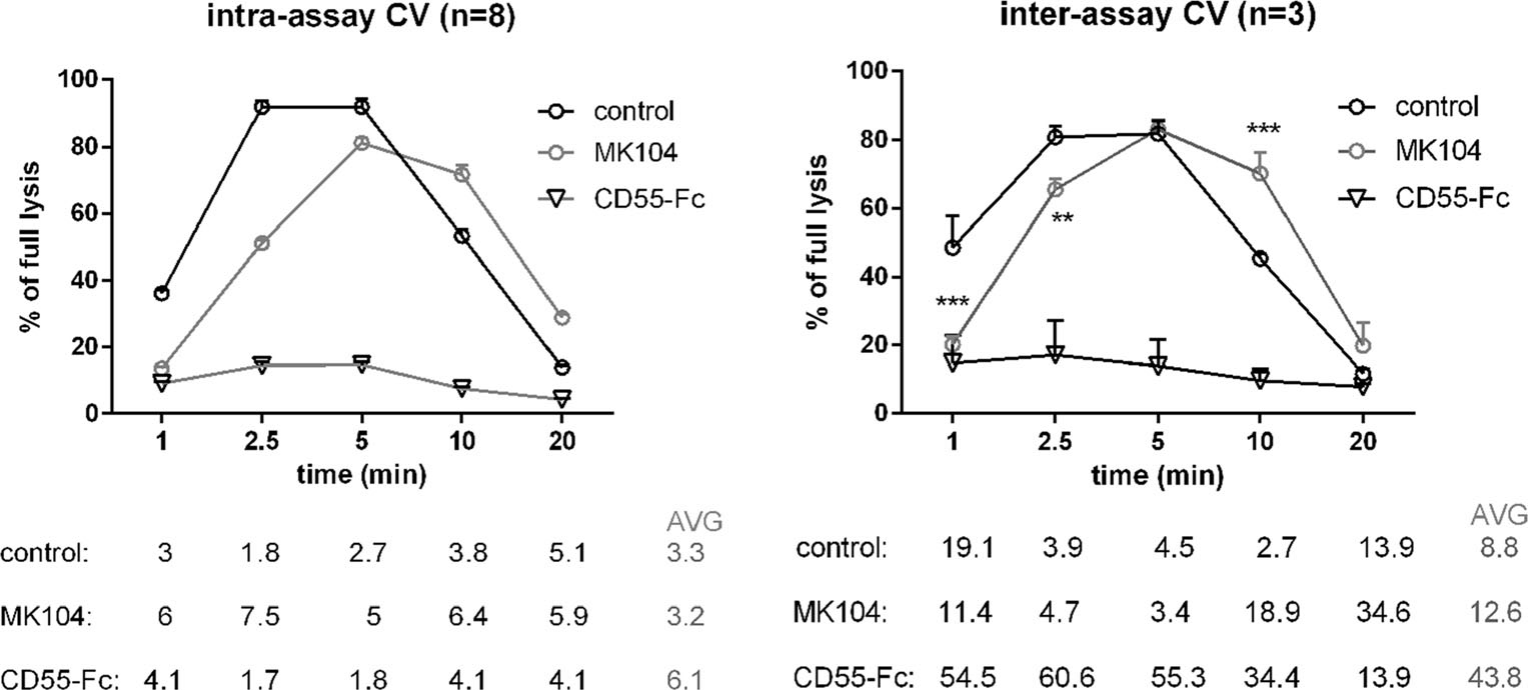
Reproducibility of the assay testing the activity of classical complement convertases. Intra- (*left panel*) and inter- (*right panel*) assay coefficient of variation were assessed for control sample (1 % NHS), positive control sample (1 % NHS supplemented with 300 nM MK104), and negative control sample (1 % NHS supplemented with 300 nM CD55-Fc). CV values for each time point are given in percent under the graphs, and average values are indicated with *gray font*. Statistical significance was assessed with two-way ANOVA assay at **p < 0.01 and ***p < 0.001

We propose that our assay may be used as an efficient screening method for presence of factors interfering with activity of classical convertases. In order to evaluate the reproducibility of the assay, we assessed intra- and inter-assay coefficient of variation (CV). Such analyses were performed for 1 % normal human serum sample expected to reflect normal profile of classical convertases activity over the time. In order to additionally validate the method and to visualize the effect of negative and positive controls, we also analyzed 1 % serum supplemented with 300 nM of soluble CD55-Fc (modeling the influence of convertase inhibitors and resulting in diminished convertase activity) and serum supplemented with 300 nM of MK104 (monoclonal antibody, which blocks function of C4BP, the main fluid-phase inhibitor of classical convertases [34]) resembling the effect of C4NeF. Intra-assay CV was performed with eight repetitions per experiment, which represented whole column of standard, 96-well microplate whereas inter-assay CV was calculated from three independent experiments (Fig. 4). Intra-assay CV values did not exceed 8 % for any of time points. Average inter-assay CV values obtained for control and MK104 samples were 8.8 and 12.6 %, respectively. However, higher CV values were obtained only for samples representing low readout. This was evident for CD55-Fc samples, which generally achieved very low readouts but variability at such close-to-background level within separate experiments resulted in substantial CV values. Nonetheless, statistical significance for difference between CD55-Fc and control samples was achieved and we assume that our method offers a good chance to distinguish samples with hyperactive classical convertases. Importantly, experiments on intra- and inter-assay CV were performed on separate batches of erythrocytes from two different vendors and obtaining of similar patterns of convertase activity additionally ensure the reproducibility of the method.

## Discussion

Convertases are central enzymatic complexes in the complement system, as they catalyze C3 and C5 activation, produce anaphylatoxins, and initiate the terminal lytic pathway. Therefore, it is not surprising that the majority of endogenous inhibitors including factor H, factor I, C4BP, CR1, CD46, and CD55 control convertase activity [35, 36] and that pushing the balance between convertase activation and inhibition by either mutations or autoantibodies results in autoinflammatory events. Certain complement inhibitors like factor H possess multiple polymorphic variants but, paradoxically, different mutations causing redundant phenotypes in terms of complement regulatory function do not predispose to the same disease [37]. This is partially explained by interactions with carbohydrates, which form distinct patterns depending on their locations, e.g., the brain, eye, or kidney [38]. However, the matter seems to be more complex and one of the acknowledged theories assumes the need of multiple hits by gain-of-function mutations in complement activators, loss-of-function mutations in complement inhibitors, and/or presence of autoantibodies for complete penetrance of diseases such as aHUS [39, 40]. The kidney is the organ that is especially vulnerable to complement-mediated damage, as on the one hand it filters substantial amount of blood under high pressure and on the other hand only a single layer, flat epithelium separates the glomerular structures from the bloodstream. It is known that C3NeF may be a single factor sufficient to cause dense deposit disease or C3 glomerulonephritis. In contrast to uncontrolled amplification of the alternative pathway as a causative factor of autoinflammatory diseases, understanding a role for C4NeF as a sole causative factor is more difficult. Certain hints may be obtained from the C4NeF cases reported in SLE [14], an autoimmune disease associated with occurrence of autoantibodies targeting DNA and nuclear antigens [41]. Importantly, the possible repertoire of targets for autoantibodies is much broader, including membrane proteins and extracellular matrix proteins [41, 42]. It is highly possible that a certain pool of autoantibodies exists in healthy individuals but their effector functions are blocked by anti-idiotypic antibodies [43, 44] or, alternatively, they are present at low titers, which are not relevant for massive complement activation. However, the appearance of C4NeF may disturb this equilibrium and push complement activation to a pathological extent.

**Fig. 5:**
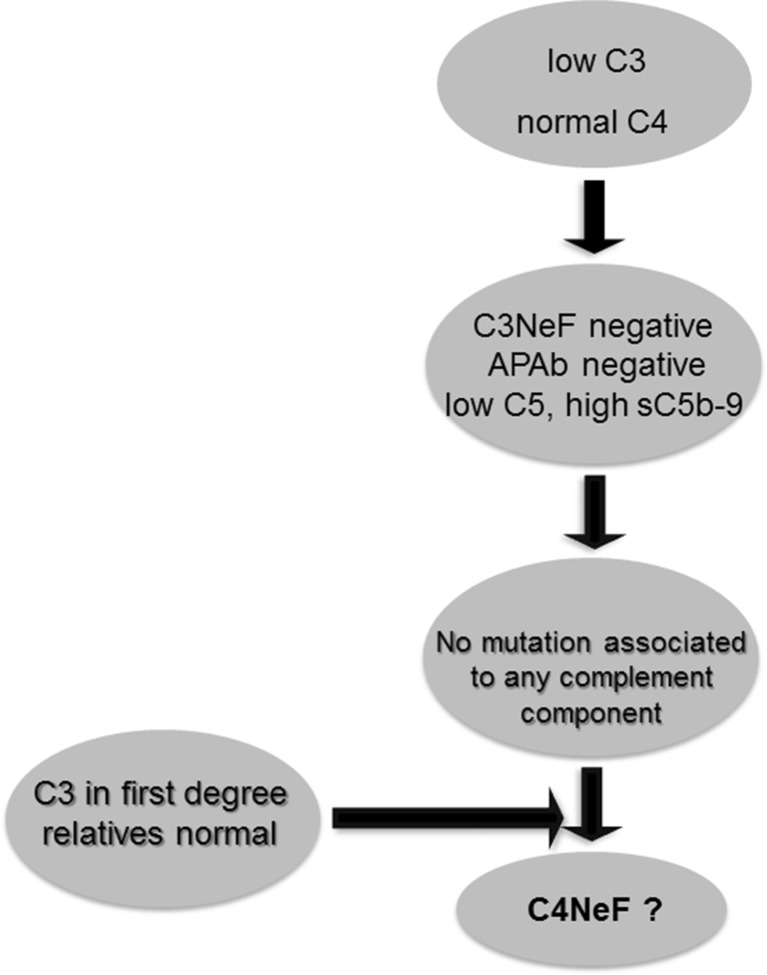
Algorithm for selection of patients with hyperactive classical convertases. *APAb* antibodies against alternative pathway components

In the present work, we report a patient who presented with hypocomplementemia, but no risk factors other than C4NeF could be identified in spite of detailed investigation. Since this is not the only reported case showing that C4NeF may exist independently from other factors or autoantibodies causing dysfunction of the complement system [13, 15, 16], our finding emphasizes the importance of screening for abnormal activity of classical convertases in clinical practice. The assay we propose is superior to other methods, as measurement of convertase activity in serum or diluted plasma takes into account all interactions, which may be physiologically relevant. Otherwise, putative masking effects of other plasma proteins or possible antagonisms or synergies taking place in individual samples may be lost and the obtained results may be over- or underestimated. Our results show that presence of C4NeF may be the only pathogenic factor found in C3G patients. Given that C4NeF is a rare phenomenon, it would be useful to implement an algorithm for selection of patients, which should be further screened for factors affecting the activity of classical convertases. One would expect that C4NeF should deplete available pool of C3 and C5, leave C4 level unaffected, and increase production of sC5b-9. On the other hand, autoantibodies targeting the alternative complement pathway as well as mutations in complement components would act as confounding factors for C4NeF. Figure 5 presents a scheme of deduction aimed to select patients with hyperactive classical convertases.

We attempted to make an insight into the nature of C4NeF identified in patient seven and found that the Ig fraction was capable of preventing the classical convertases from either spontaneous or inhibitor-driven decay, as evidenced by usage of CD55 as a model complement inhibitor with decay-accelerating function [45]. Also, we showed that, similar to another reported case of C4NeF [15], the stabilization effect was exerted for both C3 and C5 classical convertases, but in contrast to a report by Gigli et al. [12], we did not observe protection from proteolytic inactivation of C4b. This speaks for heterogeneity of C4NeF and suggests that these autoantibodies are not monospecific but target multiple epitopes on classical convertases, depending on the donor. Possible associations with diseases and/or severity are still to come once more cases of C4NeF will be functionally characterized.

## Electronic supplementary material

Below is the link to the electronic supplementary material.

Supplementary Fig. 1 Experiment was performed as in Fig. 1 but all 13 patients are presented in the graph. (GIF 38 kb)

High Resolution Image (TIFF 2006 kb)

Supplementary Fig. 2 Experiment was performed as in Fig. 1 but influence of addition of patient’s 7 plasma was compared to the addition of the same volume of total Ig fraction isolated from patient’s plasma. Data are collected from three independent experiments. (GIF 21 kb)

High Resolution Image (TIFF 835 kb)

## Abbreviations

C3NeF: C3 nephritic factor
C4NeF: C4 nephritic factor
C3G: C3 glomerulopathy
C3GN: C3 glomerulonephritis
DDD: Dense deposit disease
aHUS: Atypical hemolytic uremic syndrome
Ig: Immunoglobulin
NHS: Normal human serum
FB: Factor B
FD: Factor D
BSA: Bovine serum albumin
HRP: Horseradish peroxidase
SLE: Systemic lupus erythematous
SDS-PAGE: Polyacrylamide gel electrophoresis with sodium dodecyl sulfate
PVDF: Polyvinylidene fluoride
