# Supplementary figures and images for: Testing the Activity of Complement Convertases in Serum/Plasma for Diagnosis of C4NeF-Mediated C3 Glomerulonephritis
Source: J Clin Immunol. 2016 May 5;36:517–27. doi: 10.1007/s10875-016-0290-5 (PMC4896984; doi:10.1007/s10875-016-0290-5)

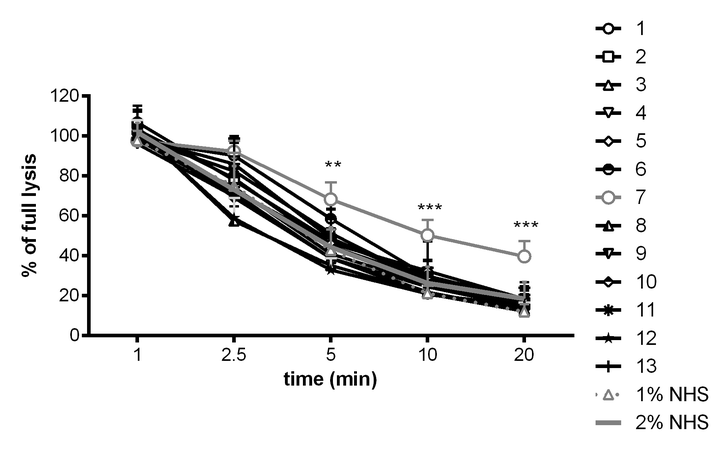

Supplement: Supplementary file 1 — Experiment was performed as in Fig. 1 but all 13 patients are presented in the graph. (GIF 38 kb) [file 10875_2016_290_Fig6_ESM.gif]

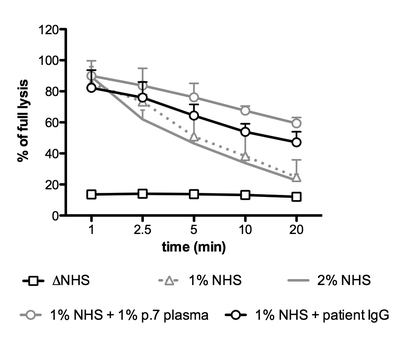

Supplement: Supplementary file 3 — Experiment was performed as in Fig. 1 but influence of addition of patient’s 7 plasma was compared to the addition of the same volume of total Ig fraction isolated from patient’s plasma. Data are collected from three independent experiments. (GIF 21 kb) [file 10875_2016_290_Fig7_ESM.gif]

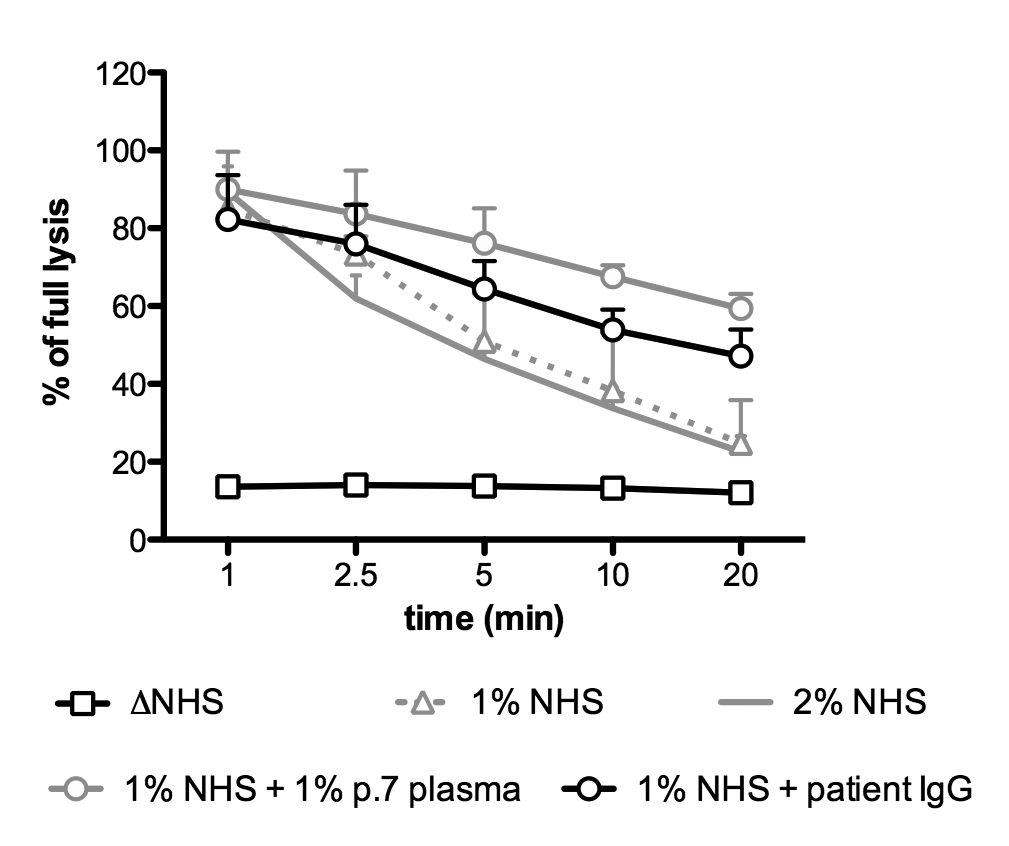

Supplement: Supplementary file 4 — High Resolution Image (TIFF 835 kb) [file 10875_2016_290_MOESM2_ESM.tiff]
